# Real-time optimized biofeedback utilizing sport techniques (ROBUST): a study protocol for a randomized controlled trial

**DOI:** 10.1186/s12891-017-1436-1

**Published:** 2017-02-07

**Authors:** Jeffrey B. Taylor, Anh-Dung Nguyen, Mark V. Paterno, Bin Huang, Kevin R. Ford

**Affiliations:** 1 Department of Physical Therapy, Congdon School of Health Sciences, High Point University, High Point, NC 27268 USA; 2 Department of Athletic Training, Congdon School of Health Sciences, High Point University, High Point, NC USA; 3 Division of Occupational Therapy and Physical Therapy, Division of Sports Medicine, Department of Pediatrics, Cincinnati Children’s Hospital Medical Center, University of Cincinnati School of Medicine, Cincinnati, OH USA; 4 Division of Biostatistics and Epidemiology; Department of Pediatrics, Cincinnati Children’s Hospital Medical Center, University of Cincinnati School of Medicine, Cincinnati, OH USA

**Keywords:** Biofeedback, Hip extensor, Knee abduction, Anterior cruciate ligament, Injury prevention

## Abstract

**Background:**

Anterior cruciate ligament (ACL) injuries in female athletes lead to a variety of short- and long-term physical, financial, and psychosocial ramifications. While dedicated injury prevention training programs have shown promise, ACL injury rates remain high as implementation has not become widespread. Conventional prevention programs use a combination of resistance, plyometric, balance and agility training to improve high-risk biomechanics and reduce the risk of injury. While many of these programs focus on reducing knee abduction load and posture during dynamic activity, targeting hip extensor strength and utilization may be more efficacious, as it is theorized to be an underlying mechanism of injury in adolescent female athletes. Biofeedback training may complement traditional preventive training, but has not been widely studied in connection with ACL injuries. We hypothesize that biofeedback may be needed to maximize the effectiveness of neuromuscular prophylactic interventions, and that hip-focused biofeedback will improve lower extremity biomechanics to a larger extent than knee-focused biofeedback during dynamic sport-specific tasks and long-term movement strategies.

**Methods:**

This is an assessor-blind, randomized control trial of 150 adolescent competitive female (9–19 years) soccer players. Each participant receives 3x/week neuromuscular preventive training and 1x/week biofeedback, the mode depending on their randomization to one of 3 biofeedback groups (hip-focused, knee-focused, sham). The primary aim is to assess the impact of biofeedback training on knee abduction moments (the primary biomechanical predictor of future ACL injury) during double-leg landings, single-leg landings, and unplanned cutting. Testing will occur immediately before the training intervention, immediately after the training intervention, and 6 months after the training intervention to assess the long-term retention of modified biomechanics. Secondary aims will assess performance changes, including hip and core strength, power, and agility, and the extent to which maturation effects biofeedback efficacy.

**Discussion:**

The results of the Real-time Optimized Biofeedback Utilizing Sport Techniques (ROBUST) trial will help complement current preventive training and may lead to clinician-friendly methods of biofeedback to incorporate into widespread training practices.

**Trial registration:**

Date of publication in ClinicalTrials.gov: 20/04/2016. ClinicalTrials.gov Identifier: NCT02754700.

## Background

Injuries in female athletes are a major short and long-term individual and public health problem within the United States. The combination of a greater susceptibility to injury than male athletes and a 10-fold increase in the female sports population since the inception of Title IX has resulted in a dramatic increase in the incidence of anterior cruciate ligament (ACL) injuries in females [1]. In the United States, 100,000–250,000 ACL injuries occur each year [2, 3]. The costs exceed $650 million annually in female varsity athletics alone [4]. In addition, there is a strong association between ACL injury and the development of posttraumatic knee osteoarthritis at a relatively young age, which also occurs with much greater incidence in females than males [5, 6]. Thus, the long-term ramifications of such injuries are quite significant. Poor athlete compliance and the absence of widespread implementation of effective ACL injury prevention programs has resulted in a consistently higher risk of ACL injury in females despite considerable research in the field.

ACL injury prevention programs use various neuromuscular training modalities to modify the high-risk biomechanics associated with injury [7]. Past research indicates that high knee abduction moments (KAM) may be the most important movement strategy to target. Hewett et al. [8] prospectively evaluated adolescent female athletes prior to their athletic season and reported KAM to be the single best predictor of subsequent ACL injury. KAM has also been reported to be significantly higher in females compared to males during a variety of landing and pivoting movements [9–14]. Furthermore, females show significant increases in KAM following rapid growth during adolescence compared to males [13]. As such, sex differences in KAM may partly explain the drastic differences in injury rates between post-pubertal males and females. Additionally, lower extremity valgus alignments are often demonstrated by females at the time of injury [15–17]. There is relative consensus in the literature that approximately 70%–80% of ACL injuries are non-contact in nature [17–19]. Video analysis techniques have confirmed that most non-contact ACL injuries occur during a sharp deceleration or landing maneuver with the knee close to extension at initial ground contact [17]. Olsen et al. performed a videographic examination of ACL injury mechanisms in team handball and reported that the ACL injury mechanism in women was a forceful valgus collapse with the knee close to full extension, combined with tibial rotation [15]. These analyses demonstrate relatively common mechanisms, including valgus, extended knee and widened stance [18].

While these previous investigations provide an important understanding of the potential mechanics related to injury, it may be more relevant to define the inciting mechanisms that underlie the high injury risk mechanics to provide the potential to target modifiable contributors to injury. Imbalances in hip function may be potential factors related to lower extremity and ACL injuries in female athletes [20, 21], considering that males utilize 18% greater hip extensor moments during the concentric phase of landing compared to females [22]. Additionally, sex differences are present in hip joint posture at initial contact (greater flexion in males), peak internal hip extensor moment (greater magnitude in males) and a significant preference to underutilize the hip compared to the knee extensors [23], indicating that males utilize a different hip strategy during landing compared to females. The hip extensors (gluteus maximus, hamstrings) are not only important in extending from a squat position but also in eccentrically establishing or maintaining posture and balance when landing from a jump. The targeting of proximal mechanisms, notably the activation and strength of the hip extensors, may result in improved control of knee abduction load and posture, ultimately leading to a greater reduction in the risk of ACL injury in young female athletes [24].

Prophylactic neuromuscular training has been shown to increase active knee stabilization in the laboratory and decrease the incidence of ACL injury on the field and court of play in athletic female populations [2, 25–28]. Neuromuscular training facilitates neuromuscular adaptations that focus on joint stabilization and safe movement patterns. This training allows female athletes to adopt muscular recruitment strategies that decrease joint motion and protect the ACL from high impulse loading [26, 29]. However, inconsistent implementation [7], combined with low athlete compliance [30] has resulted in a lack of wide-spread reduction in ACL incidence [1] and an alarming increase in ACL reconstruction surgeries in females and patients younger than 20 [31].

Despite the lack of wide-spread reduction in ACL injuries, numerous components of neuromuscular training have proven beneficial in modifying high-risk biomechanics and decreasing injury risk. Technique feedback has been reported to be beneficial for athletes to improve biomechanics and has become a recommended component of ACL injury prevention practices [32]. Conventional ACL injury prevention programs utilize verbal feedback from clinicians, coaches, and/or teammates to correct mechanics such as the position of the athlete’s knees. However, the type of cuing may be very important, as instructional strategies emphasizing an external-focus may be more beneficial for retention and the transfer of learned biomechanics to sport activities than internally-focused cues [33]. More specifically, biofeedback has been successful in retraining the biomechanics of runners [34], but has yet to be incorporated in ACL injury prevention programs, despite a pilot study reporting that kinetic biofeedback given during squats rapidly transfers to dynamic drop landings [35]. Thus, further trials studying the methodology, implementation and efficacy of biofeedback as a complement to traditional neuromuscular based prevention programs is warranted.

This paper describes the design of the Real-time Optimized Biofeedback Utilizing Sport Techniques (ROBUST) trial, the first study attempting to describe the immediate effects and retention of specific neuromuscular movement training using biomechanical biofeedback in order to reduce the risk of ACL injuries in adolescent female athletes. The trial is designed to assess the effectiveness of biofeedback as a complement to traditional neuromuscular prophylactic training and to identify whether feedback targeting the risk of injury (knee abduction load) or an underlying mechanism of injury (underutilization of the hip musculature) is more beneficial in this athletic population. Given the relatively high-risk of injury in female athletes, the grueling process of surgery and rehabilitation, and the impending early development of osteoarthritis, this trial has the potential to positively impact current preventive strategies. This manuscript will further explain in detail the injury prevention training program, biofeedback methodology, and screening of biomechanical outcome variables of this randomized control trial.

### Hypotheses

Our central hypothesis is that biofeedback methodology will maximize the effectiveness of neuromuscular prophylactic interventions. More specifically, we hypothesize that ROBUST training (both knee- and hip-focused) will lead to significantly reduced knee abduction load during double-leg jump landings, but that hip-focused ROBUST training will significantly reduce knee abduction load during a high-risk unplanned cutting task compared to knee-focused training. Further, we hypothesize that participants that receive hip-focused ROBUST training will best retain improvements in knee abduction load six months following training.

## Methods/design

### Study design

**Fig. 1:**
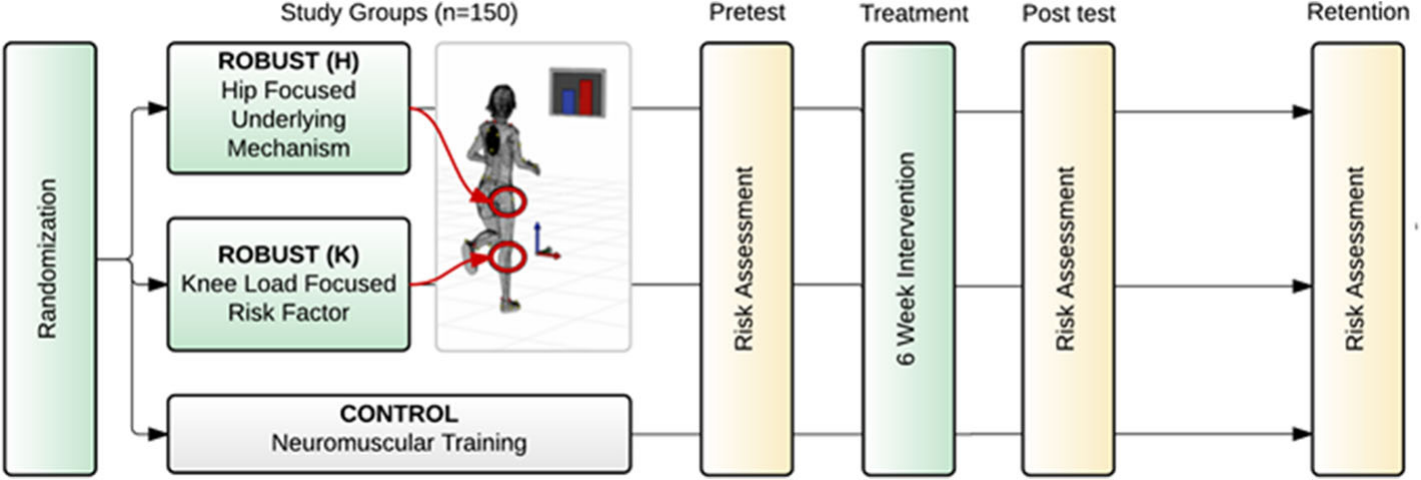
Flow diagram of study participants randomized into three arms of ROBUST trial: ROBUST (H): Hip-focused biofeedback, ROBUST (K): Knee-focused biofeedback, and CONTROL: Sham biofeedback

ROBUST is a prospective, randomized, active comparator, open blinded, end-point trial. Participants will be randomized into one of three study arms 1) hip-focused biofeedback, 2) knee-focused biofeedback, and 3) sham biofeedback. Each participant will provide written participant consent, and/or parental consent and participant assent as appropriate. All participants will be tested immediately before (pre-test), immediately after (post-test), and 6 months after (retention) a 6-week prophylactic neuromuscular training program. The training program will be performed 3 times a week, with augmented biofeedback (according to group designation) 1 time a week. All testing and training will occur at the High Point University Human Biomechanics and Physiology Laboratory. Figure 1 provides a flowchart of the design of ROBUST. Researchers responsible for collecting the primary outcome measures will be blinded to the participant’s group designation. While participants randomized to the study arms of the trial will not be blinded, we will also make every effort to maintain the blinding of the researchers responsible for delivering the interventions. In addition, the investigators and biostatistician will remain blinded to the group status during data management and analysis. In case a participant needs to be treated for an injury during the study period, the treating clinicians will not be aware of the group assignment. In addition, teams consisting of a trainer and blinded research assistants will be maintained as separate, functioning teams throughout the study to minimize the chances of unblinding. The study protocol was approved by the Institutional Review Board at High Point University. Additional information can be found at: Clinicaltrials.gov (Identifier: NCT02754700).

### Participants

One hundred fifty female soccer players between the ages of 9 and 19 will be recruited from local soccer clubs and high schools for this study. Recruitment will be performed primarily through discussions with club presidents, athletic directors, directors of coaching, coaches, and parents. The study sample will consist solely of female youth soccer players that participate on a competitive team at the time of enrollment. Participants will be excluded if they are not currently able to participate in sport due to an injury or cannot commit to participating in the 6-week intervention.

An equal sample size of *N* = 50 will be randomized to each of the three study arms. Assuming 20% of loss to follow up, a sample size of 40 is expected in each group at the conclusion of the study. Under this assumption, it was estimated that one-way analyses of variance will have 80% of power to detect at the 0.05 level a difference in means characterized by a variance of means of 0.082 (i.e. the averaged group mean deviations from the grand mean being 0.082 or higher of the common standard deviation). Previous results suggest an improvement in knee abduction load between two forms of biofeedback training with estimated mean+/-SD of 11+/-6 Nm vs. 2+/-10 Nm, indicating sufficient statistical power in this study [35].

### Randomization

Enrolled subjects will be randomized to a biofeedback group using computer-generated blocks after completing pre-test screening. A clinical coordinator, independent of the study statistician and research assistants conducting the analyses, trainer, and evaluator, will generate a random sequence of numbers which will be stored in a spreadsheet accessible to only non-blinded study staff. Participants will be assigned to the study arms in the randomization scheme according to the order that their baseline visit was completed. The clinical coordinator will generically label the treatment assignments to group A, B, and C to ensure blinding is maintained for researchers responsible for pre- and post- testing, and for researchers responsible for providing the prophylactic intervention. Only the clinical coordinator and researchers responsible for providing ROBUST training will be privy to the participants’ group designation.

### Interventions

#### ACL injury prevention training

The ACL injury prevention training program will be organized into 18 total sessions over 6 consecutive weeks with a frequency of 3 times a week. The duration of each session will be 90 mins, including a 9–10 min active warm-up, and 3 separate 27–30 min sessions of resistance training, technique/plyometric training, and core strength training. All training will be overseen by a licensed athletic trainer with expertise in screening and prevention of ACL injury and amply trained undergraduate research assistants majoring in exercise science, athletic training, and/or biology. At each session, participants will complete the children’s format of the OMNI-Resistance Exercise Scale [36] immediately after finishing each training component.

The training program is based on the collective previous work of the research team and focused on previous studies that have reduced knee injuries in female athletes [7, 37]. Initial development was derived from Hewett et al. [38] and Myer et al. [4] Recommended guidelines were followed when designing the integrated, comprehensive training program [39]. The resistance training component consists of two sessions a week of hip and knee focused strengthening and one session a week of upper body and ankle strengthening. Resistance will be provided by a variety of mediums, including bodyweight, kettlebells, medicine balls, traditional weight machines, and pneumatic resistance [24]. Table 1 details the resistance training exercise progression. Both the plyometric and core strengthening components were adapted from a pilot study reported by Myer et al. [40] Within each technique/plyometric session, training begins with closed chain, body weight exercises that focus on optimal technique, with the expectation that technique translates as exercises are progressed from controlled to ballistic movements. Specific exercise progressions transition from double- to single-leg, sagittal plane to frontal and/or transverse plane, stable to unstable surfaces, and planned to unplanned movements. Table 2 describes the 6-week progression of the technique/plyometric training component. The core strengthening component focuses on improved activation, co-contraction, and strength of the abdominal, low back, proximal hip, and thigh musculature. Exercises use a variety of unstable surfaces, including BOSU® Balance Trainers and therapeutic exercise balls to perturb the body and enhance engagement of the core musculature. Specifics of the core strengthening training progression can be found in Table 3.

**Table 1:** Resistance training protocol utilized during ROBUST trial

Exercise	Method of resistance	Week 1	Week 2	Week 3	Week 4	Week 5	Week 6
*Hip and Thigh (2x/week)*							
Bench squat	BW	2 x 12	-	-	-	-	-
Goblet squat	KB	-	2 x 12	2 x 10	2 x 10	2 x 8	2 x 8
Deadlift	AIR	2 x 12	2 x 12	2 x 10	2 x 10	2 x 8	2 x 8
Stationary lunges	BW	2 x 12	2 x 12	-	-	-	-
Walking lunges	BW	-	-	2 x 10	-	-	-
Walking lunges	KB (unilateral resistance)	-	-	-	2 x 10	-	-
Walking lunges	KB (with rotation)	-	-	-	-	2 x 8	-
Walking lunges	KB (unilateral shoulder press)	-	-	-	-	-	2 x 8
Reverse hypers	Weight	2 x 12	2 x 12	2 x 10	2 x 10	2 x 8	2 x 8
Single-leg RDL	BW	2 x 12	-	-	-	-	-
Single-leg RDL	KB	-	2 x 12	2 x 10	-	-	-
Runners	BW	-	-	-	2 x 10	-	-
Runners	KB	-	-	-	-	2 x 8	2 x 8
Band walk	RB (at knees)	2 x 15	-	-	-	-	-
Band walk	RB (at ankles)	-	2 x 15	-	-	-	-
Band walk	RB (at toes)	-	-	2 x 15	-	-	-
X Band monster walk	RB (at shoulders)	-	-	-	2 x 15	-	-
X Band monster walk	RB (W’s)	-	-	-	-	2 x 15	-
X Band monster walk	RB (overhead)	-	-	-	-	-	2 x 15
*Upper Extremity and Ankle (1x/week)*							
Seated cable row	Weight	2 x 12	2 x 12	-	-	-	-
Bent over row	DB	-	-	2 x 10	2 x 10	-	-
Reverse pull-ups	BW	-	-	-	-	2 x 8	2 x 8
Push-ups	BW (wide stance on knees)	2 x 15	-	-	-	-	-
Push-ups	BW (narrow stance on knees)	-	2 x 15	-	-	-	-
Push-ups	BW (wide stance on toes)	-	-	2 x 15	-	-	-
Push-ups	BW (narrow stance on toes)	-	-	-	2 x 15	2 x 15	2 x 15
Shoulder press	KB	2 x 12	2 x 12	2 x 10	2 x 10	2 x 8	2 x 8
Triceps dips	BW	2 x 12	2 x 12	2 x 10	2 x 10	2 x 8	2 x 8
Ankle circuit	RB	2 x 12	2 x 12	2 x 10	2 x 10	2 x 8	2 x 8

*BW* body weight, *KB* kettlebell, *AIR* pneumatic resistance, *RB* resistance band, *RDL* Romanian deadlift

**Table 2:** Technique and plyometric training protocol utilized during ROBUST trial (3x/week)

Exercise	Week 1	Week 2	Week 3	Week 4	Week 5	Week 6
Double leg squat	1 x 8	1 x 8	1 x 8	1 x 8	1 x 8	1 x 8
Wall jumps	15 sec	15 sec	15 sec	-	-	-
180^o^ wall jumps	-	-	-	15 sec	15 sec	15 sec
Squat jumps	10 sec	10 sec	15 sec	-	-	-
Squat-tuck jump	-	-	-	10 sec	12 sec	15 sec
Broad jump hold	1 x 8	1 x 8	-	-	-	-
Broad jump-jump-jump-hold	-	-	1 x 6	-	-	-
Broad jump-vertical hold	-	-	-	1 x 6	-	-
Broad jump-vertical reaction	-	-	-	-	1 x 6	-
Broad jump-jump-jump vertical reaction	-	-	-	-	-	1 x 8
Box drop deep hold	1 x 10	1 x 10	-	-	-	-
Box drop vertical	-	-	1 x 10	-	-	-
Box drop broad jump hold	-	-	-	1 x 10	-	-
Box drop 180^o^ vertical hold	-	-	-	-	1 x 8	-
Box drop 180^o^ vertical reaction	-	-	-	-	-	1 x 8
Lateral jump and hold	1 x 8	1 x 8	-	-	-	-
Lateral jumps	-	-	10 sec	-	-	-
Lateral hop and hold	-	-	-	1 x 8	-	-
Lateral hops	-	-	-	-	10 sec	-
X-hops	-	-	-	-	-	6 cycles
Single leg squat	1 x 5	1 x 5	1 x 5	1 x 5	1 x 5	1 x 5
Step-hold	1 x 8	1 x 8	-	-	-	-
Jump-single leg hold	-	-	1 x 8	-	-	-
Hop-hold	-	-	-	1 x 8	-	-
Hop-hop hold	-	-	-	-	1 x 8	-
Crossover-hop-hop-hold	-	-	-	-	-	1 x 10
Single-leg lateral Airex hop-hold	1 x 4	1 x 4	-	-	-	-
Single-leg lateral Bosu hop-hold	-	-	1 x 8	-	-	-
Single-leg lateral Bosu hop-hold with catch	-	-	-	1 x 4	-	-
Single-leg 4-way Bosu hop-hold	-	-	-	-	3 cycles	-
Single-leg 4-way Bosu hop-hold with catch	-	-	-	-	-	20 sec
Single tuck jump-soft landing	1 x 10	1 x 10	-	-	-	-
Double tuck jump	-	-	1 x 6	-	-	-
Repeated tuck jump	-	-	-	10 sec	-	-
Side-to-side barrier tuck jumps	-	-	-	-	10 sec	-
Side-to-side reaction barrier tuck jumps	-	-	-	-	-	10 sec
Lunge jumps	10 sec	10 sec	-	-	-	-
Scissor jumps	-	-	10 sec	-	-	-
Lunge jumps (unilaterally weighted)	-	-	-	10 sec	-	-
Scissor jumps (unilaterally weighted)	-	-	-	-	10 sec	-
Scissor jumps with ball swivel	-	-	-	-	-	1 x 10
Single-leg 90^o^ hop-hold	1 x 8	1 x 8	-	-	-	-
Single-leg 90^o^ Airex hop-hold	-	-	1 x 8	-	-	-
Single-leg 90^o^ Airex hop-hold reaction catch	-	-	-	1 x 8	-	-
Single-leg 180^o^ Airex hop-hold	-	-	-	-	1 x 8	-
Single-leg 180^o^ Airex hop-hold reaction catch	-	-	-	-		1 x 8

**Table 3:** Core strength training protocol utilized during ROBUST trial (3x/week)

Exercise	Week 1	Week 2	Week 3	Week 4	Week 5	Week 6
BOSU supermans	1 x 12	1 x 12	-	-	-	-
BOSU supermans with perturbation	-	-	1 x 10	-	-	-
Prone bridge (elbows and knees) hip extension opposite shoulder flexion	-	-	-	1 x 10	-	-
Prone bridge (elbows and toes) hip extension	-	-	-	-	1 x 10	-
Prone bridge (elbows and toes) hip extension opposite shoulder flexion	-	-	-	-	-	1 x 10
BOSU double knee-hold	20 sec	20 sec	-	-	-	-
BOSU single knee-hold	-	-	20 sec	-	-	-
Swiss ball double knee-hold	-	-	-	20 sec	-	-
Swiss ball double knee-hold with perturbation	-	-	-	-	20 sec	-
Swiss ball double knee-hold with catch	-	-	-	-	-	20 sec
BOSU double leg pelvic bridges	1 x 10	1 x 10	-	-	-	-
BOSU single leg pelvic bridges	-	-	1 x 10	1 x 10	-	-
BOSU single leg pelvic bridges with ball hold	-	-	-	-	1 x 10	-
Supine swiss ball hamstrings curl	-	-	-	-	-	1 x 10
BOSU lateral crunch	1 x 10	1 x 10	-	-	-	-
Box lateral crunch	-	-	1 x 10	-	-	-
BOSU lateral crunch with catch	-	-	-	1 x 8	-	-
Swiss ball lateral crunch	-	-	-	-	1 x 15	-
Swiss ball lateral crunch with catch	-	-	-	-	-	1 x 8
Box double crunch	1 x 15	1 x 15	-	-	-	-
Box swivel double crunch	-	-	1 x 15	-	-	-
BOSU swivel ball touches (feet up)	-	-	-	1 x 15	-	-
BOSU double crunch	-	-	-	-	1 x 15	-
BOSU swivel double crunch	-	-	-	-	-	1 x 15
Swiss ball back hyperextensions	1 x 15	1 x 15	-	-	-	-
Swiss ball back hyperextensions and reach	-	-	1 x 15	-	-	-
Swiss ball back hyperextensions with back fly	-	-	-	1 x 15	-	-
Swiss ball back hyperextensions with reach lateral	-	-	-	-	1 x 15	-
Swiss ball back hyperextensions with lateral catch	-	-	-	-	-	1 x 15
Russian hamstrings curl	1 x 10	1 x 10	1 x 10	1 x 10	1 x 10	-
Swivel Russian hamstrings curl	-	-	-	-	-	1 x 10

### ROBUST

Subjects will participate in a biofeedback session (10 min) once weekly over the course of the training program. A three-dimensional motion analysis system, consisting of fourteen digital high-resolution cameras (Raptor-12, Motion Analysis Corporation, Santa Rosa, CA), and two time-synchronized, embedded, oversized force platforms (AMTI, Watertown, MA) will be used to provide biofeedback for participants. For each ROBUST session, participants will be instrumented with retroreflective markers bilaterally on their medial and lateral malleoli, medial and lateral knee joint line, ASIS, greater trochanter, and the sacrum and left PSIS to define the ankle (centroid method), knee (centroid method) and hip joint centers (Bell method) respectively. Additionally, rigid 4-marker clusters will be affixed bilaterally to each participant’s lateral thigh and shank with cohesive tape (Pro-Trainer® cohesive tape, Medco Sports Medicine^TM^, Tonawanda, NY) and four individual markers will be placed on the foot (toe, anterior lateral foot, posterior lateral foot, heel) with double-sided tape for tracking purposes. After instrumentation, a static trial will be collected of the participant in anatomical position to model the segment coordinate system and define each tracking maker. The static model will be defined directly in Visual3D with the model used directly during real-time biofeedback.

**Fig. 2:**
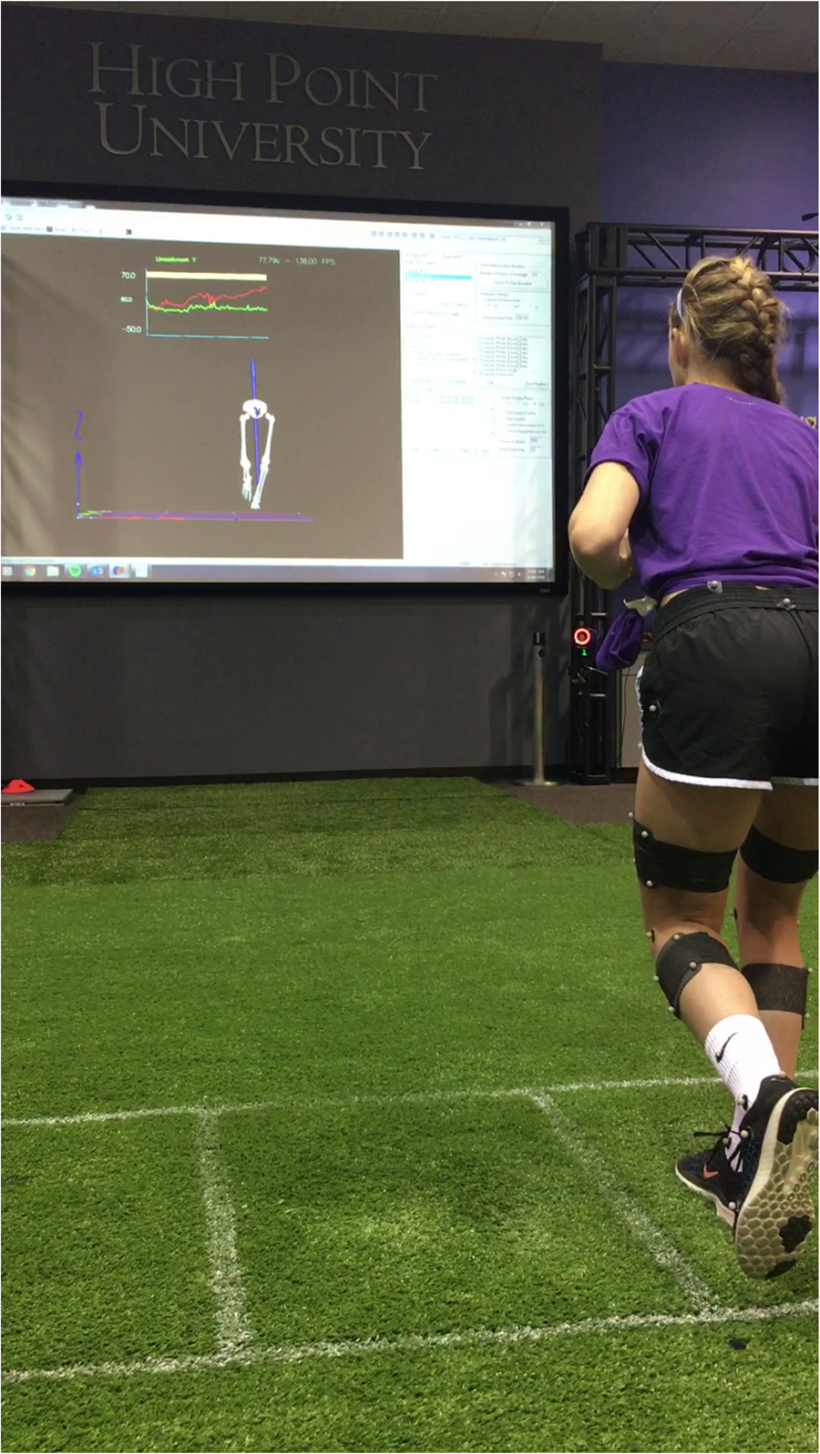
Sample participant during biofeedback training

For ROBUST training, each participant will be in front of a 95 ft^2^ screen which will depict a real-time avatar and graph of either hip extensor moment (hip-focused group), knee abduction moment (knee-focused group), or knee flexion angle (sham group) with a highlighted goal region that they are encouraged to attain (Fig. 2). At each session, participants will perform a series of 3 exercises (Table 4) including a double-leg squat, single-leg squat, and single-leg jump landing. Although encouraged to focus on the visual biofeedback data, participants will receive verbal instructions and feedback from a member of the research team. Members of the hip-focused group will be instructed to activate posterior-chain muscles throughout the movements in order to increase the hip extensor moment feedback. The knee-focused group will be instructed to maintain knees over toes while pushing laterally through their feet. Participants that receive sham biofeedback are instructed to bend the knees while squatting.

**Table 4:** Exercises performed during ROBUST biofeedback sessions

Weeks 1 -2	Week 3	Week 4	Week 5	Week 6
Double leg squat (DLS)	DLS arms front	DLS arms chest	DLS arms up	DLS deep
Single leg squat (SLS)	SLS arms front	SLS arms chest	SLS arms up	SLS deep
Step hold	Jump single leg hold	Hop hold	Hop hop hold	Crossover hop hop hold

### Outcome measures

At the pre-testing session, all participants will complete an electronic RedCap (Research Electronic Data Capture) [41] survey detailing their 1) demographic information, 2) sport participation history, 3) lower extremity and spine injury history, 4) parental heights for calculation of pubertal stage, and 5) menstrual history.

The primary outcome variables of interest for this study will include lower extremity biomechanics during double- and single-leg jump landing and unplanned cutting tasks. Participants will perform these tasks on a synthetic turf (TurfLink TL80), while wearing standardized cleats (adidas x15.2; Beaverton, Oregon, USA). Each participant will be instrumented for 3-dimensional biomechanical analysis with 43 retroreflective markers placed on the sternum, sacrum, left posterior superior iliac spine, C7, 3 points on the upper back (via a thin backpack), and bilaterally on the shoulder, upper arm, elbow, wrist, anterior superior iliac spine, greater trochanter, midthigh, medial and lateral knee joint line, tibial tubercle, midshank, distal shank, medial and lateral malleolus, and to the foot at the heel, dorsal surface of the lateral midfoot, lateral rear foot and toe via double-sided tape. A static trial will be collected to determine each subject’s neutral alignment and anatomically define each body segment, by which subsequent biomechanical measures will be referenced. Using Cortex software (version 5; Motion Analysis Corp, Santa Rosa, California, USA) three-dimensional motion capture will be sampled at 200 Hz and kinetic data will be sampled at 1200 Hz with the system previously described.

### Landing biomechanics

**Fig. 3:**
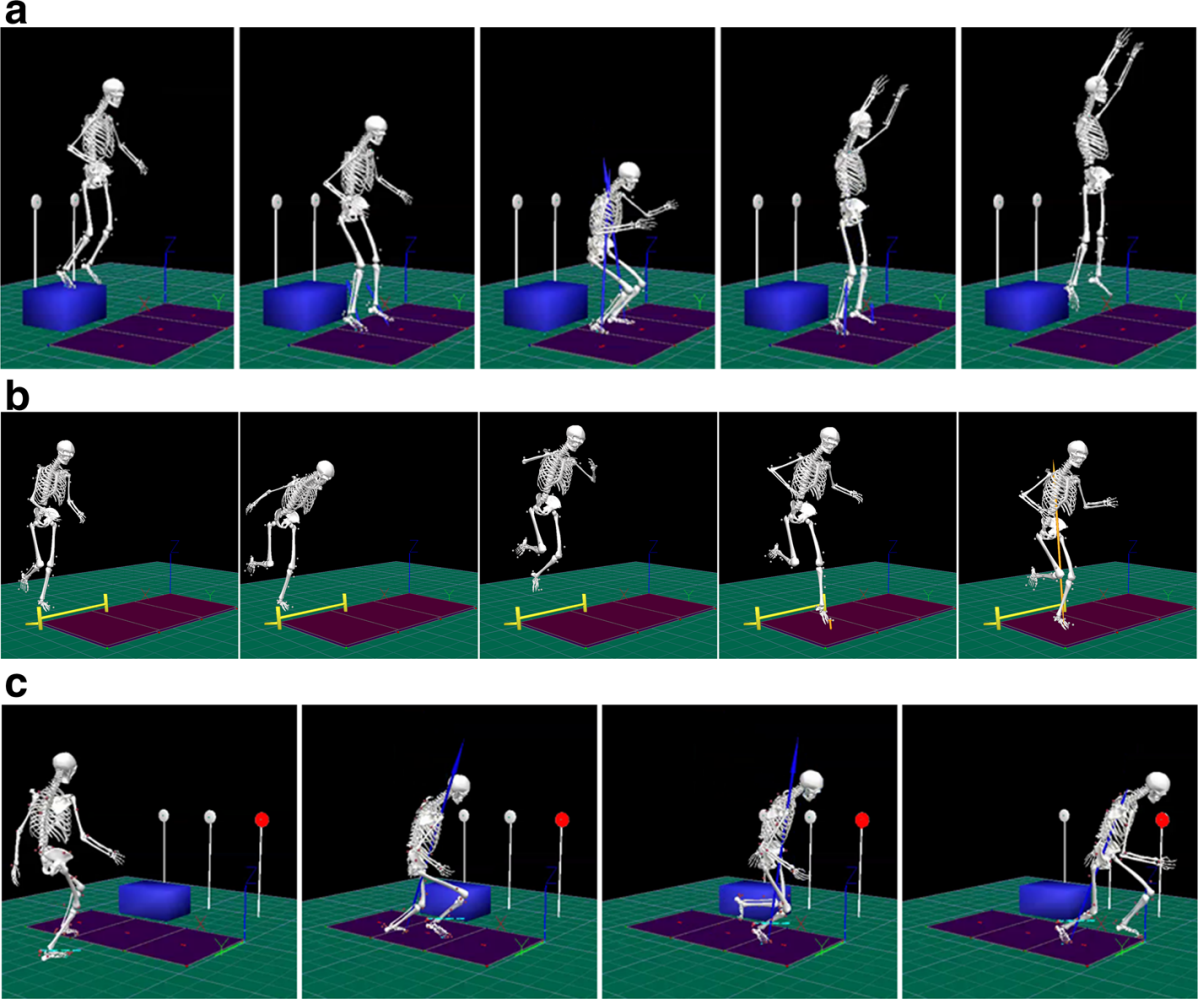
Knee abduction moments during (**a**) double-leg landings, (**b**) single-leg landings, and (**c**) unanticipated cutting will be the primary outcome measures

Each participant will perform three trials of a drop vertical jump (DVJ) (Fig. 3a). Participants will start on top of a 31-cm box, with their feet positioned 35-cm apart and arms at their side. A member of the research team will instruct them to drop down directly off the box, land on both feet at the same time and immediately perform a maximal vertical jump, reaching for an overhead target that was previously placed at their maximal vertical countermovement jump reach.

Participants will then perform three trials of a single-leg landing (Fig. 3b) on each limb in a previously randomized order, and the order will be identical for post- and 6 month retention testing sessions. A meter stick will be placed adjacent to the landing area, beginning at a target placed in the center of the force plate. A starting line will be placed on the meter stick at 40% of the subject’s height. A 5-inch hurdle will be placed midway between the starting line and landing target. Participants will be instructed to stand on one leg with their toe at the starting line. They will then hop forward over the hurdle on one leg, land on the same leg and hold the landing for 2 s without allowing the non-stance limb to touch the ground or come in contact with the stance limb. An inability to hold the landing for 2 s will result in repeating the trial.

### Cutting biomechanics

For the unplanned cutting task, each participant will complete 3 successful trials of a 90° sidestep cut and backpedal cut on each limb, with the order of cuts and backpedals randomized for each limb (Fig. 3c). Prior to beginning the trial, participants are made aware of which limb they will be cutting or backpedaling off. For example, if the right limb is the limb of interest, participants will either plant on their right limb and cut 90° to the left or make initial contact with their right limb to decelerate and backpedal. Participants will start 5 m away from the force plates and be instructed to run forward at 75% of their maximal speed. A trigger will be placed 2 m in front of the force plate that, when passed, will immediately illuminate a light (FITLIGHT trainers™, FITLIGHT Sports Corp., Aurora, Ontario, Canada) placed 1.5 m behind the force plate telling the athlete to cut (light placed at waist level towards the cutting direction) or backpedal (light placed at eye level directly behind of the force plates). As stated, speed will be self-selected, though participants will be encouraged to maintain 75% of their maximal speed, and the speed of each trial will be measured using two timing gates (TracTronix, Lenexa, Kansas, USA), placed 2.5 m apart, with the latter gate 0.45 m from the front edge of the force plate. Trials will be repeated if the participant cuts or backpedals off the wrong limb, or does not get their plant limb fully on to the force plate when cutting.

### Secondary outcome measures

**Fig. 4:**
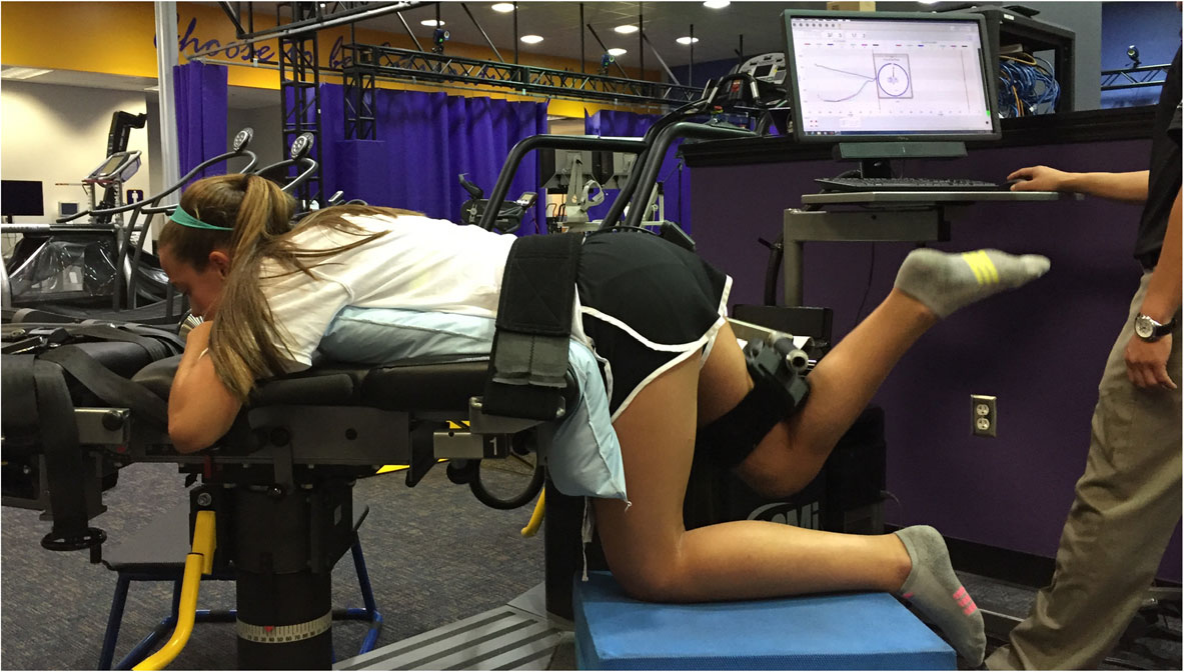
Participants’ isokinetic concentric and eccentric hip extensor strength will be tested at pre-, post-, and retention testing sessions

Isokinetic hip extensor strength will be tested using an isokinetic dynamometer (HUMAC/NORM Testing and Rehabilitation System, Computer Sports Medicine Inc. Stoughton, Massachusetts, USA) at a speed of 60° per second (Fig. 4). Participants will be placed in the prone position with their anterior inferior iliac spines on the table, their hip and knee flexed to 90°, and their greater trochanter aligned with the rotational axis of the dynamometer. A stabilization strap will be placed over the pelvis to ensure that the participant’s hips stay on the table throughout the trials. The resistance pad of the dynamometer will be positioned at the distal thigh, just proximal to the popliteal fossa. To un-weight the contralateral limb, a box will be placed underneath the knee of the contralateral limb for the participant to rest with their hip flexed to 90°. Both concentric and eccentric hip extensor strength will be measured between 90 and 30° of hip range of motion in conjunction with verbal encouragement from the tester. Each participant will complete 3 practice repetitions prior to completing 5 maximal effort repetitions for each condition. The limb and contraction condition (eccentric or concentric) will be randomized for each participant. Peak torque and work per repetition will be calculated for the second, third, and fourth repetitions.

Double-leg vertical and single-leg horizontal jump performance will be measured during a countermovement jump (CMJ) and single-leg hop for distance, respectively. CMJ height will be captured during biomechanical testing with the 3D motion capture system on the synthetic turf surface. Participants will complete three maximal effort trials while reaching their hands vertically towards an overhead target that was placed at the tips of the fingers during 2–3 CMJ practice trials. Jump height will be calculated as the difference between the peak vertical displacement of the center of mass and the position of the center of mass during static standing. Participants will also complete three trials of a single-leg hop for distance on each limb while wearing a standardized sneaker (adidas adipure 360.2, Portland, OR, USA) on a rubber gym floor. While standing on one leg with their toes behind the starting line, participants will leap forward as far as possible, landing on the same limb and holding the landing for 2 s. The researcher will measure the distance from the starting line to the distance of the toe using a tape measure affixed to the floor. Trials will be repeated if the participant is unable to hold the landing for 2 s and/or touches the ground or the hopping leg with the opposing limb.

Quickness and change of direction performance will be measured during three trials of the pro-agility drill [42]. Participants will stand in the middle of a single timing gate (SMARTSPEED™, Fusion Sport Inc., Australia) placed in the center of a court surface. When the light on the timing gate flashes green, participants are instructed to turn and sprint in one direction (self-selected) to a piece of tape on the floor 4.57 m away, turn and sprint 9.14 m in the opposite direction to another piece of tape on the floor, turn and sprint 4.57 m until they pass through the timing gate. The total time to complete the trial will be recorded for analysis.

Core muscle strength will be assessed using the sports-specific core muscle test as previously described by Tong et al. [43] Participants will begin in a standard prone plank position on their forearms and toes, while attempting to hold for one minute. If able, participants will subsequently transition through the following stages of a prone plank 1) lifting right arm off the ground and holding straight ahead for 15 s, 2) lifting left arm off the ground and holding straight ahead for 15 s, 3) lifting right leg off the ground for 15 s, 4) lifting left leg off the floor for 15 s, 5) lifting both right arm and left leg off the floor for 15 s, 6) lifting left arm and right leg off the floor for 15 s, 7) standard prone plank for 30 s, 8) repeating progression from step 1 above. Participants will complete one trial of this test, which is measured in total time by the researcher with a standard stop watch. Trials will be stopped if participants cannot maintain a proper plank position (i.e. hip elevation, drop, or shift) following a maximum of 3 verbal corrections in each stage or if a body part other than the participants’ forearms and toes makes contact with the ground.

### Retention testing

After completing the 6-week training program and post-test assessment, participants will provide monthly sport participation, athletic exposure, and injury history data. An electronic survey will be emailed to the participants and their families to help with accurate data collection and retention efforts. Participants will return to the Human Biomechanics and Physiology Laboratory 6 months after training, utilizing the same researchers and baseline methodology.

### Data and statistical analysis

Intention-to-treat (ITT) analysis will be performed according to CONSORT statement guidelines [44]. Baseline differences between groups will be analyzed using an analysis of variance (ANOVA). Improvement in the biomechanical outcomes of knee load and hip-strategy will be assessed using Paired-*T* test (when normal) or Signed Wilcoxon rank-sum test (when non-normal) for each study arm. An ANOVA will be used to compare the improvement in post- vs. pre-training in knee load and the hip-strategies across the three study arms. Tukey’s multiple comparison procedures will be used to adjust for multiple comparisons. ANOVA analyses will consider adjusting for age and other baseline covariates as determined earlier. All ITT participants will be analyzed. Further, we will adopt multivariate ANOVA techniques, which will be modeling the study outcome measures as a whole in testing study hypotheses.

### Timeline

Human subjects review board approval was obtained in November, 2015 from the High Point University Institutional Review Board. Recruitment and training began in June 2016. A projected 75 subjects will be enrolled in the study within the first year, followed by 75 participants in the 2^nd^ year. Final data collection is planned to be completed by June, 2018 and final analyses to be completed by April, 2019.

## Discussion

Prophylactic neuromuscular training can reduce the risk of ACL injury [7]; however, ACL injury rates continue to remain high [1]. Continuing to improve and refine these programs is necessary to improve widespread implementation and reduce the risk of this debilitating injury that can lead to an arduous rehabilitation process, reduced likelihood of returning to pre-injury level of activity, high re-injury rates and the potential for the early onset of osteoarthritis. Early signs point to biofeedback as an effective complement to traditional neuromuscular training as a method of providing externally-focused feedback to adolescent female athletes at risk for injury [35]. This trial will elucidate the benefits of biofeedback and help ascertain whether providing feedback of the biomechanical variable most predictive of injury (KAM) or the underlying mechanism of abnormal biomechanics (hip extensor moment) is most beneficial for these athletes as they transition from controlled training to dynamic, sport-specific activities. Further, this study will better help understand the long-term effects of prophylactic neuromuscular training and the extent to which these modes of feedback may better help with the retention of modified biomechanics.

This trial will be the first randomized control trial to evaluate the potential benefits of biofeedback with traditional neuromuscular training. In itself, the training is evidence-based yet novel, as it incorporates a combination of resistance, plyometric, and core training in a high-intensity off-season program. Many current programs have transitioned to warm-up programs, utilizing 15–25 min before practice for technique training at relatively low intensities [7]. However, while many have been successful at reducing injury risk, these programs that use general strength and plyometric exercises may not provide a large enough stimulus to promote lasting long-term biomechanical modifications to improve and maintain athlete safety [45–47]. Our 90-min program provides a progression of high-intensity exercises that especially target the posterior chain musculature (i.e. gluteals, hamstrings). Additionally, this study will complement past research on biomechanical changes throughout maturation [48–51], as 9–19 year olds will be recruited to participate. The stage at which biomechanical modifications occur and the extent to which prophylactic training and biofeedback elicit these modifications will be able to be teased out.

While an impactful clinical trial, the ROBUST trial does have some minor limitations. As this study is designed to optimize prevention interventions, the study design does not include a traditional control group as other biomechanical studies of neuromuscular preventive training have done. As stated previously, evidence indicates that neuromuscular training is beneficial at reducing injury risk. Thus, our study design is focused on the identification of optimal interventions and the decision was made to train each participant and group participants based on the type of biofeedback provided. As such, the control group in our study will receive a “sham” condition focused on a kinematic variable that is expected to have little consequence on biomechanics since a previous study reported significantly improved outcomes with kinetic compared to kinematic feedback [35]. This variable will also not affect the extent of the task as all participants will be instructed to obtain the same amount of center of mass displacement during the tasks. Additionally, the target for the sham condition will remain the same throughout the training to control the extent of the task while the target for the hip- and knee-focused groups will be progressed each session.

Given the significant short- and long-term physical, economic and psychosocial ramifications of an ACL injury in adolescent female athletes, improving the efficacy of ACL injury prevention programs is vital. Results of this study will uncover the best methods of biofeedback for improved biomechanics to transfer to sport-specific dynamic tasks and retain improvements throughout the course of a season. Future studies could lead to the transition of laboratory-based biofeedback to on-field biofeedback through wearable technology, ultimately impacting biomechanics where it matters most.

## Abbreviations

ACL: Anterior cruciate ligament
ANOVA: Analysis of variance
ASIS: Anterior superior iliac spine
Cm: Centimeter
CMJ: Countermovement jump
DVJ: Drop vertical jump
Hz: Hertz
ITT: Intention-to-treat
KAM: Knee abduction moment
Nm: Newton*meters
PSIS: Posterior superior iliac spine
ROBUST: Real-time optimized biofeedback utilizing sport techniques (ROBUST)
